# The functional diversity of essential genes required for mammalian cardiac development

**DOI:** 10.1002/dvg.22794

**Published:** 2014-06-24

**Authors:** Christopher Clowes, Michael GS Boylan, Liam A Ridge, Emma Barnes, Jayne A Wright, Kathryn E Hentges

**Affiliations:** 1 Faculty of Life Sciences, University of Manchester, Michael Smith BuildingOxford Road, Manchester, United Kingdom; 2 Department of Product Safety, Syngenta LtdJeallots Hill, United Kingdom

**Keywords:** heart, sarcomere, trabeculation, cardiac chamber specification, epicardium, heart fields

## Abstract

Genes required for an organism to develop to maturity (for which no other gene can compensate) are considered essential. The continuing functional annotation of the mouse genome has enabled the identification of many essential genes required for specific developmental processes including cardiac development. Patterns are now emerging regarding the functional nature of genes required at specific points throughout gestation. Essential genes required for development beyond cardiac progenitor cell migration and induction include a small and functionally homogenous group encoding transcription factors, ligands and receptors. Actions of core cardiogenic transcription factors from the Gata, Nkx, Mef, Hand, and Tbx families trigger a marked expansion in the functional diversity of essential genes from midgestation onwards. As the embryo grows in size and complexity, genes required to maintain a functional heartbeat and to provide muscular strength and regulate blood flow are well represented. These essential genes regulate further specialization and polarization of cell types along with proliferative, migratory, adhesive, contractile, and structural processes. The identification of patterns regarding the functional nature of essential genes across numerous developmental systems may aid prediction of further essential genes and those important to development and/or progression of disease. genesis 52:713–737, 2014.

## INTRODUCTION

Alteration or deletion of genes is a valuable methodology to determine gene function. The tools available to modern molecular biologists have rendered the removal of genes a systematic process as reflected by the current availability of increasing numbers of targeted deletions and conditional alleles (Dolgin, 2011; Skarnes *et al*., 2011). Additionally, chemical mutagenesis particularly in combination with modern sequencing approaches continues to provide an invaluable resource in attempts to complete the functional annotation of the genome (Arnold *et al*., 2011; Brown *et al*., 2013; Gondo *et al*., 2010; Kile *et al*., 2003; Oliver and Davies, 2012; Probst and Justice, 2010). The ease of performing genetic manipulations in the mouse and the early availability of its genomic sequence led to its emergence as the mammalian model of choice for functional studies (Waterston *et al*., 2002). As functional data accumulates, it is now possible to identify which individual genes and biological functions are indispensible for the progression of specific processes. Genes absolutely required for an organism to develop to maturity and for which there is no compensation for critical aspects of their function are considered essential. The proportion of all mouse genes that are essential for embryonic development as well as their genomic distribution/density and the roles of their human orthologs in human diseases are among questions that are being actively pursued (Dickerson *et al*., 2011; Georgi *et al*., 2013; Hentges *et al*., 2007; Wilson *et al*., 2005). Less consideration has been paid to the functional diversity of genes that are essential to specific developmental processes and the temporal requirements for differential functions. We argue that at different timepoints in cardiac specification, the functional diversity of essential genes changes, with different functions becoming more or less represented in cardiac essential genes.

Many genes involved in mammalian cardiac development are essential, due to the requirement for cardiac function in utero early during gestation. Cardiac development involves the spatially and temporally coordinated actions of individual cells, tissues, and regulated gene expression for cell recruitment, differentiation, and organ morphogenesis (Bruneau, 2002; Buckingham *et al*., 2005; Conway *et al*., 2003; DeRuiter *et al*., 1992; Dunwoodie, 2007; Vincent and Buckingham, 2010; Wagner and Siddiqui, 2007a, 2007b; Zaffran and Frasch, 2002). Embryonic lethality has been shown to result from the altered function of a wide range of genes contributing to cardiac development. In this review, cardiac development will be analyzed from a temporal and structural perspective. Examples of genes essential to cardiac development at different stages or in specific processes will be detailed, and their roles analyzed to present a composite of the functional diversity of cardiac essential genes. Some of the processes required in cardiogenesis are needed in embryogenesis in general, so cardiac essential genes may also affect the development of the early embryo or other organ systems. This review predominantly covers genes and genetic pathways specific to cardiogenesis.

## CARDIAC INDUCTION AND MIGRATION

**Figure 1:**
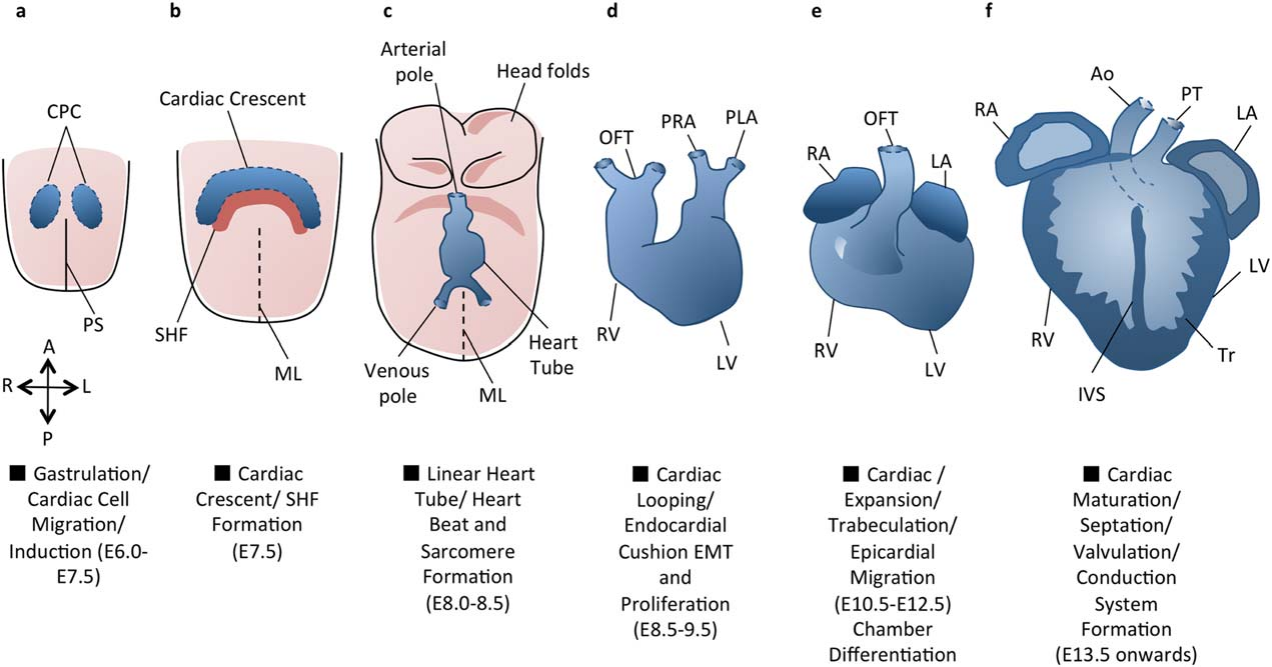
An overview of cardiac development. Cardiac development progresses from the specification of cardiac progenitor cells (a) to the migration of these cells towards the midline to form the cardiac crescent (b). The developing heart then forms a linear tube (c), which undergoes dextral looping to acquire the appropriate left–right asymmetry (d). The heart tube is further subdivided into the four chambers (e), and the maturation of the endocardial cushions into the valves and development of the great vessels provides for unidirectional blood flow through the chambers (f). Adapted from (Buckingham *et al*., 2005). A = Anterior, Ao = Aorta, CPC = Cardiac Precursor Cells, IVS = Interventricular septum, L = Left, LA = Left Atrium, LV = Left Ventricle, ML = Midline, OFT = Outflow Tract, P = Posterior, PHF = Primary Heart Field, PLA = Primitive Left Atrium, PRA = Primitive Right Atrium, PS = Primitive Streak, PT = Pulmonary Trunk, R = Right, RA = Right Atrium, RV = Right Ventricle, SHF = Secondary Heart Field, Tr = Trabeculae.

During the first stages of cardiac development, after gastrulation, at embryonic day (E) 6.5–7.5, cardiac progenitors from the anterior mesodermal primitive streak migrate anteriorly and laterally on either side of the embryonic midline (Fig. 1A) (Tam and Behringer, 1997; Tam *et al*., 1997); primary heart field (PHF) cells are derived from these cells. The PHF cells then migrate medially, form the epithelial cardiac crescent (Fig. 1B) and begin to differentiate in situ before fusing to form the linear heart tube (Fig. 1C) (Bruneau, 2002; Buckingham *et al*., 2005; Dunwoodie, 2007; Wagner and Siddiqui, 2007b; Zaffran and Frasch, 2002). In addition to intrinsic signals from within the primitive streak, cardiac cell migration and induction depends on signaling from the anterior lateral mesoderm, anterior endoderm and nonneural ectoderm during gastrulation and crescent formation. These signals regulate cardiac induction while defining the mediolateral borders of the heart-forming region (Arai *et al*., 1997; Auda-Boucher *et al*., 2000; Dunwoodie, 2007).

During gastrulation, anterior primitive streak cells transiently express the basic helix-loop-helix family (bHLH) transcription factor *Mesp1*, recognized as the first detectable cardiac marker (David *et al*., 2008; Kitajima *et al*., 2000; Saga, 1998; Saga *et al*., 2000; Saga *et al*., 1999). *Mesp1* expressing cells are incorporated into all mature cardiac layers (i.e., endothelium, endocardium, myocardium and epicardium) (Saga *et al*., 2000). *Mesp1* knockout mice display lethality by E10.5 due to cardia bifida through failure of the linear heart tube to fuse (Saga *et al*., 1999). *Mesp1* and *Mesp2* double knockouts, however, display complete migratory block of cardiac precursors and do not develop mesoderm-derived structures including the heart, somites or gut (Kitajima *et al*., 2000). This indicates that Mesp1 and Mesp2 may be able to compensate for the lack of each other to some extent. Mesp1 may also be critical in promoting differentiation of cells that contribute to the cardiovascular system including cardiomyocytes, endothelial, and smooth muscle cells (Bondue *et al*., 2008; Bondue *et al*., 2011; David *et al*., 2008; Lindsley *et al*., 2008).

Bone morphogenetic proteins (BMPs) predominantly promote cardiac specification in the PHF. The specifics of BMP signal transduction have been considered in greater detail elsewhere (van Wijk *et al*., 2007; Wang *et al*., 2011). However, several ligands from this family are essential to cardiac development. *Bmp2* null mutant mice experience embryonic lethality between E7.0 and E10.5 with severely delayed, ectopic or absence of cardiac development (Zhang and Bradley, 1996). *Bmp4* null mice similarly demonstrate lethality from E6.5 to E9.5 where the majority of null embryos lack mesoderm-derived structures; those that do achieve the initiation of the heartbeat die soon thereafter with widespread and severe developmental delays (Winnier *et al*., 1995). Unsurprisingly, BMP receptor null mice such as *Alk3*, *Alk2*, and *Bmpr2* mutants mirror these phenotypes with lethality occurring by E9.5 due to absence of mesoderm-derived structures (Beppu *et al*., 2000; Gu *et al*., 1999; Mishina *et al*., 1995).

Fibroblast growth factor (Fgf) function is also required for cardiac development. *Fgf8* knockout mice demonstrate failure of mesodermal cell migration from the primitive streak during gastrulation at ∼E7.0 and lack mesoderm-derived and endoderm-derived tissues, including the heart, despite cells undergoing epithelial-to-mesenchymal transition (EMT) (Sun *et al*., 1999). The cardiac abnormalities found in *Fgf8* mutants are mirrored by *Fgfr1* null mice (Deng *et al*., 1994). There is considereable crosstalk between the BMP and FGF signaling pathways in chick models; FGF signaling maintains a pool of undifferentiated stem cells. For cardiomyocyte induction to occur, BMP signaling must downregulate the FGF pathway (Hutson *et al*., 2010; Tirosh-Finkel *et al*., 2010). It is not unreasonable to propose that a similar crosstalk mechanism occurs in mice.

## HEART FIELDS AND CARDIAC PROGENITOR CONTRIBUTIONS

The mutant phenotypes described above are associated with the absence of PHF cell contributions and failure of PHF cell programming towards a cardiogenic fate. After its formation, the linear heart tube grows through cellular contributions at the arterial and venous poles (Buckingham *et al*., 2005; Vincent and Buckingham, 2010; Viragh and Challice, 1973). This was originally thought to be entirely from the PHF, however, *LacZ* transgene expression under *Fgf10* promoter control in mice revealed contributions of a second heart field (SHF) to the outflow tract (OFT) myocardium, with transgene expression originating in the pharyngeal mesoderm from E7.5 (Kelly *et al*., 2001). The SHF was found to lie anteriorly and dorsally to the PHF before migrating caudally and medially to the cardiac crescent and then dorsally to the linear heart tube (Fig. 1B). DiI labeling and retrospective clonal analysis later demonstrated SHF contributions to the OFT, the majority of the right ventricle and parts of the atria. PHF cells were found to contribute to the entire left ventricle, the majority of both atria and parts of the right ventricle (Meilhac *et al*., 2004; Zaffran *et al*., 2004). Later contributions of cardiac progenitor cells come from the proepicardial organ (PEO), which will be discussed in more detail below.

Contributions of the SHF to myocardial and endocardial cell populations have been shown in vivo and SHF progenitor cells demonstrate differentiation into myocardial, endocardial and smooth muscle cells in vitro (Moretti *et al*., 2006; Verzi *et al*., 2005). The LIM- and homeodomain-containing transcription factor *Islet1 (Isl1)* is vital to SHF migration, survival, and differentiation. Mice null for *Isl1* display absence of SHF derived structures (OFT and right ventricle) and have severely reduced atrial tissue (Cai *et al*., 2003; Lin *et al*., 2007); they experience developmental arrest by ∼E9.5 and lethality around E10.5. Isl1 activity in the SHF is at least partly mediated by forkhead transcription factors such as *Foxc1* and *Foxc2* that bind SHF-specific enhancer sequences within the *Isl1* locus to direct *Isl1* expression (Kang *et al*., 2009). Mice null for *Foxc1* or *Foxc2* and heterozygous null/wildtype for the other gene experience lethality from E12.0 to E12.5; the *Foxc2* null mice particularly had a shortened OFT and smaller right ventricle (Seo and Kume, 2006). However, double knockout mice exhibit lethality by E9.5 with a complete absence of the OFT and right ventricle, indicating that *Foxc1* and *Foxc2* are partially functionally redundant (Seo and Kume, 2006). Similar phenotypes were observed in *Foxh1* null embryos (von Both *et al*., 2004). As the SHF has not yet been genetically defined, additional genes essential for and specific to SHF development likely await discovery.

## CHARACTERISTICS OF ESSENTIAL GENES IN EARLY CARDIAC DEVELOPMENT

Essential genes that cause lethality early in cardiac development, disrupting the processes of induction, migration of cardiac progenitors to the cardiac crescent, and heart field formation, represent a small and functionally homogeneous group with respect to their biochemical function. This group of genes consists predominantly of transcription factors, ligands and receptors. The lack of functional diversity at this stage of heart development is unsurprising given that the early-gestational embryonic heart represents a relatively homogenous cell population. Conversely, some genes essential to cardiac development are essential to other earlier developmental processes. Fgf and Bmp signaling pathway members both frequently demonstrate multiple developmental requirements, with null alleles exhibiting lethality during gastrulation, although these genes have later cardiac functions (Arman *et al*., 1998; Marguerie *et al*., 2006; Qi *et al*., 2007; Sirard *et al*., 1998). Furthermore, the presence or absence of functional redundancy can obscure the role of some genes in early-gestational cardiac development. For example, Wnt signaling ligands possess high protein sequence homology, overlapping expression domains and overlapping functions during early cardiac development which compensates for the absence of each other (Cohen *et al*., 2008), making the dissection of their specific functions challenging. Some processes occurring during early cardiac development may only have later functional consequences. For example, cardiac looping begins at ∼E8.5 but problems resulting from loss of concordance between the heart and other organs may not cause lethality until later in development (Kim, 2011). The limited numbers of genes with essential functions specific to early cardiac developmental processes suggest a relatively restricted genetic programme required for cardiac specification and heart field formation.

## CARDIAC CHAMBER FORMATION

Positional identity is established within cardiac progenitors early during cardiac development, prior to the formation of the linear heart tube. However, regions of force-producing chamber and primary nonchamber myocardium are only formed on the onset of cardiac looping (Fig. 1D). Differentiation into atrial and ventricular chamber myocardium occurs locally on the outer curvature of the looping heart simultaneously with rapid proliferation of differentiated cells to form the chambers. During this process, cell-type specific transcriptional programmes that pattern the chambers along the left–right, anteroposterior, and dorsoventral axes also initiate the specialization of cells within the chambers. This collective process of differentiation, proliferation, and specialization is known as the ballooning model of chamber morphogenesis (Christoffels *et al*., 2004a; Christoffels *et al*., 2000; Delorme *et al*., 1997; Moorman *et al*., 2010). Cardiac cell terminal differentiation and cardiac morphogenesis are governed by a core set of essential transcription factors, particularly those from the Nkx, Gata, Mef, Hand, and Tbx families. These transcription factors act as a point of convergence for earlier, upstream pathways and act combinatorially with each other, as well as with cardiac-specific and ubiquitous transcription factors to target the expression of genes involved in later events in cardiac development (Olson, 2006). Understandably, genes from these families are indispensible for the progression of cardiac development beyond midgestation.

In cardiac development, *Gata4* has been the most extensively studied family member of Gata zinc finger-containing transcription factors. *Gata4* is expressed in the precardiac mesoderm from E7.0 before expanding to the endocardium and myocardium throughout heart tube formation and persisting through adult life (Heikinheimo *et al*., 1994). Homozygous *Gata4* deficient mice suffer embryonic lethality between E7.0 and E9.5 and fail to form the linear heart tube due to inappropriate lateral to ventral embryonic folding and extraembyonic defects (Kuo *et al*., 1997; Molkentin *et al*., 1997). Embryo-specific deletion of *Gata4* in mice also results in a range of cardiac defects including incorrect looping morphogenesis, thin myocardium with altered cytoarchitecture, reduced trabeculation, absence of the atrioventricular canal (AVC) and bulboventricular groove, absence of endocardial cushions (ECCs) and absence of the proepicardium (Watt *et al*., 2004). The range in phenotypes caused by disruption of GATA4 function is partly due to its involvement in the expression of sarcomeric genes including *Myh6*, *Myl1*, and *Tnni3* (Di Lisi *et al*., 1998; McGrew *et al*., 1996; Molkentin *et al*., 1994). Gata4 has additional roles in cardiogenesis; it interacts with Fog2 and a *Cx30.2* enhancer to promote ECC formation and the atrioventricular conduction system development, respectively (Flagg *et al*., 2007; Munshi *et al*., 2009). Furthermore, chamber expansion appears reliant on Gata family proteins as Gata4 interacts with core cell cycle machinery and cooperates with Gata5 to regulate cardiomyocyte proliferation (Rojas *et al*., 2008; Singh *et al*., 2010; Trivedi *et al*., 2010). Thus, Gata4 acts at both transcriptional and posttranscriptional levels with several other transcription factors and DNA regulatory elements in a cardiac cell-type specific manner (Brown *et al*., 2004; Lien *et al*., 1999; Lozano-Velasco *et al*., 2011; Maitra *et al*., 2009; Munshi *et al*., 2009; Sepulveda *et al*., 1998; Sepulveda *et al*., 2002; Singh *et al*., 2010).

The homeodomain*-*containing transcription factor Nkx2–5 is another key regulator and essential gene during cardiac development. Nkx2–5 forms complex regulatory loops with Gata4 and lies both upstream and downstream of Gata4 in different systems, although its expression is more spatially restricted than *Gata4* within the heart (Brown *et al*., 2004; Riazi *et al*., 2009). *Nkx2–5* is expressed in cardiac progenitors within the mesoderm during mouse development from E7.5 before being present in myocardial cells throughout cardiac development and adult life (Kasahara *et al*., 1998; Lints *et al*., 1993). *Nkx2–5* null mice experience lethality between E9.5 and E11.5 due to subsets of abnormalities including cardiac looping defects, abnormal OFT development, absent ECCs, reduced trabeculation, shortened AVC, absence of one ventricle and lack of cardiomyocyte differentiation (Lyons *et al*., 1995; Tanaka *et al*., 1999). In addition to cardiomyocyte differentiation, Nkx2–5 appears essential for differentiation of endocardium, components of the conduction system, epicardium and formation of the AVC (Ferdous *et al*., 2009; Habets *et al*., 2002; Jamali *et al*., 2001; Moskowitz *et al*., 2007; Prall *et al*., 2007; Zhou *et al*., 2008b). Again, this is unsurprising given that Nkx2–5 acts upstream of numerous critical genes expressed during cardiac development such as *Npr1*, *Ankrd1*, *Cx40*, *Actc1*, and *Myocd* (Bruneau *et al*., 2001; Chen and Schwartz, 1996; Shiojima *et al*., 1999; Ueyama *et al*., 2003; Zou *et al*., 1997). Additionally, Nkx2–5 is central to pathways controlling cardiac cell proliferation (Prall *et al*., 2007; Qi *et al*., 2007; Zhang *et al*., 2010b). These cellular and morphological events are collectively mediated through differential interactions with cardiac-specific transcription factors and DNA regulatory elements in a cell-type specific manner (Chen and Schwartz, 1996; Puskaric *et al*., 2010; Sepulveda *et al*., 2002; Shiojima *et al*., 1999; Zou *et al*., 1997). Given the influence of Gata4 and Nkx2–5, many midgestational lethal cardiac phenotypes demonstrate aberrant signaling pathways associated with Gata4 and Nkx2–5.

The MADS-box transcription factor Mef2c acts immediately downstream of and cooperates with Nkx2–5 and Gata4 in cardiac development (Dodou *et al*., 2004; Skerjanc *et al*., 1998; Vincentz *et al*., 2008). *Mef2c* expression is first detected in the precardiac mesoderm at E7.5, then in the common atrium and ventricle of the heart tube and throughout the myocardium of all chambers during midgestation before declining to lower level expression in late-gestation to adult life (Edmondson *et al*., 1994; Naya *et al*., 1999). *Mef2c* null mice suffer lethality between E9.5 and E10.5 due to inability to undergo cardiac looping, absence of the right ventricle, atrial and ventricular hypoplasia, reduced trabeculation and shortened AVC with absent ECCs. Like Gata4 and Nkx2–5, Mef2c appears to be essential for cardiomyocyte differentiation in vivo (Karamboulas *et al*., 2006). Several genes have also been identified whose cardiac expression is dependent on upstream signaling of Mef2c including *Calr*, *Tnni3k*, *Ctnna3*, *Champ*, and *Smyd1* (Liu *et al*., 2001; Phan *et al*., 2005; Qiu and Michalak, 2009; Vanpoucke *et al*., 2004; Wang *et al*., 2008). Lineage tracing analyses have demonstrated contributions of *Mef2c* expressing cells to the ventricles and atrioventricular bundle and a role for Mef2c in allocating cells of PHF origin to ventricular or sinoatrial node fate (Aanhaanen *et al*., 2010; Vong *et al*., 2006). Mef2c has also been suggested to have specific SHF roles downstream of Isl1 and Gata4 (Dodou *et al*., 2004).

Cardiac chamber formation is achieved by dividing the developing heart into different functional compartments (Fig. 1E). The *heart and neural crest derivatives expressed transcript* (*Hand)* genes are among the earliest differentially expressed genes during cardiac development. The *Hand1* and *Hand2* bHLH family transcription factors are both uniformly expressed in mice at E7.75 during the cardiac crescent stage of heart development before becoming restricted to left and right ventricles, respectively, during and after cardiac looping (Srivastava *et al*., 1997; Thomas *et al*., 1998). *Hand2* null mouse embryos suffer lethality around E11.0 due to absence of a right ventricle and lack of aortic arch arteries (Srivastava *et al*., 1997). Rescue of extraembryonic defects by tetraploid aggregation in *Hand1* null mice (to circumvent peri-implantation lethality) demonstrated cardiac defects including defective looping and failure to establish distinct atria and ventricles and lack of trabeculation (Riley *et al*., 1998). The critical roles of Hand1 are in maintenance of proliferation of cardiomyocytes. Removal of *Hand1* and *2* in a dose-dependent manner demonstrate their co-operative regulation of ventricular chamber growth and ventricular hypoplasia (McFadden *et al*., 2005). Furthermore, Hand1 has been suggested to regulate the balance between cardiomyocyte differentiation and proliferation. Mouse embryos overexpressing *Hand1* exhibit overexpansion of the linear heart tube whilst embryonic stem cells overexpressing *Hand1* upregulate cell cycle gene expression of *cyclin D2* (*Ccnd2)* and *cyclin-dependent kinase 4* (*Cdk4)*, which prevents cell cycle exit (Risebro *et al*., 2006). Ventricular growth mediated by Hand proteins depends on the regulation of myocardial gene expression, which is mostly achieved cooperatively with upstream signals from Gata4, Nkx2–5, and Mef2c (Bruneau *et al*., 2000; Dai *et al*., 2002; Thattaliyath *et al*., 2002; Yamagishi *et al*., 2001; Zang *et al*., 2004a, 2004b).

The T-box (Tbx) transcription factors represent a group of at least 18 transcription factors that have diverse roles in cardiac development. Regarding chamber development, Tbx5 plays important roles in regulating an atrial-specific transcriptional program. During development, *Tbx5* is expressed from cardiac crescent to looping heart tube stage hearts in areas destined to become the atria and sinus venosus. *Tbx5* expression then gradually expands to the left ventricle and right ventricular trabeculae, atrial septum, left aspect of the ventricular septum, and atrioventricular valves as the heart matures (Bruneau *et al*., 1999). *Tbx5* null mice suffer lethality by E10.5 due to looping abnormalities, failure to form two atria and hypoplasia of the single atrium and left ventricle (Bruneau *et al*., 2001).

*Tbx2* is essential during cardiac development with respect to nonchamber structures. *Tbx2* is expressed in the AVC and atrioventricular cushions, OFT and inflow tract in the linear and looping heart before gradually decreasing to background levels by E15.0. In these regions, Tbx2 is thought to repress chamber-specific gene expression such as *Nppa*, *Smpx*, *Cx40*, and *Slit3* allowing these areas to remain as primary myocardium for purposes of chamber alignment and to form specialist structures of ECCs, valves, septa, and conduction system components (Aanhaanen *et al*., 2011; Christoffels *et al*., 2004b; Medioni *et al*., 2010). *Tbx2* null mice suffer lethality by E14.5 demonstrating abnormal AVC morphology, severely retarded ECC growth, OFT septation defects and ectopic expression of chamber-specific genes *Nppa* and *Smpx* (Harrelson *et al*., 2004). Ventricular defects have also been demonstrated in *Tbx2* deficient embryos due to reduced contributions of cells from the primary myocardium that typically turn off *Tbx2* expression before contributing to the expanding chambers and septum (Aanhaanen *et al*., 2009). *Tbx2* expression is directly repressed by Tbx20; overexpression of *Tbx2* in the myocardium and loss of *Tbx20* generates similar cardiac phenotypes (Cai *et al*., 2005; Christoffels *et al*., 2004b). Evidence suggests that spatial regulation of primary myocardium-specific gene expression is as equally important as initiation of chamber-specific expression during chamber development.

## CHAMBER EXPANSION

After chamber identity is fixed, the myocardium expands to ensure it can pump blood around the growing embryo, while the heart responds to signaling cues directing chamber separation and orientation (Fig. 1F). While the muscle mass is growing the myocardium begins compaction from E11.5 (most markedly between E13 and E14) which contributes to formation of the interventricular septum, papillary muscle, and conduction system and maximizes the amount of muscle that can contribute to the heart. The concurrent actions of ventricular myocardial growth and compaction of the muscle itself allows for production of greater contractile force (Dunwoodie, 2007; Risebro and Riley, 2006; Wagner and Siddiqui, 2007b). Several genes that maintain cardiac chamber expansion and morphogenesis following the actions of the core cardiogenic transcription factors are essential for cardiac development.

Transcription factors are important in regulating growth of the cardiac chambers, as well as their specification. The MADS-box transcription factor Srf acts with Gata4 and Nkx2–5 to regulate chamber myocardium growth (Sepulveda *et al*., 1998). Global knockouts of Srf are early lethal (Arsenian *et al*., 1998), but cardiomyocyte, smooth muscle, and endothelial-specific knockouts of Srf all exhibit midgestation lethality with a panopoly of cardiac defects caused by defective chamber specific differentiation (Holtz and Misra, 2008; Miano *et al*., 2004; Niu *et al*., 2005; Parlakian *et al*., 2004). Tef1, a downstream effector of Srf1, has important roles in muscle-specific gene expression and recognizes regulatory sequences present in the cardiac-specific genes *Myh7*, *Myocd*, and *Acta2* (Gan *et al*., 2007; Gupta *et al*., 2001; Yoshida, 2008). Retroviral gene-trap of *Tef1* in mice results in embryonic lethality in homozygous mutants at E11.5–E12.5 with mutants exhibiting thin myocardium and decreased trabeculation (Chen *et al*., 1994).

The maintenance of mechanotransductive signals is required to support cardiac chamber morphogenesis and expansion. By midgestation genes that sense mechanical stress also become essential for cardiogenesis. G-protein coupled receptors (GPCRs) initiate cascades in response to mechanical force, controlling heart rate, and force of contraction. This response can be desensitized by agonists binding to GPCRs resulting in their subsequent phosphorylation by GPCR kinases (Hata and Koch, 2003). A fine balance must be achieved between desensitization and cascade initiation during cardiac development. Disruption of adrenergic receptor kinase *Adrbk1* in mice causes embryonic lethality due to heart failure in homozygous mutants between E9.0 and E15.5 caused by hypoplasia of the ventricular septum and all cardiac chambers, resulting in poor ejection fraction and heart failure (Jaber *et al*., 1996). Myocardial-specific ablation of *Fak*, which is needed for stretch induced Mef2 activation (Nadruz *et al*., 2005), induces defects in cardiomyocyte proliferation and ventricular septal defects (Peng *et al*., 2008). Other proteins involved in mechanotransduction, such as Erk1, Erk2, and Pxn, also display cardiac morphological defects which suggest links between blood flow, cardiac contraction, and cardiomyocyte proliferation (Granados-Riveron and Brook, 2012).

## CHARACTERISTICS OF GENES ESSENTIAL IN CHAMBER FORMATION AND EXPANSION

During the processes of cardiac chamber formation and expansion there is a great increase in the number of transcription factors required, as compared to earlier stages of cardiac development. Members of several different transcription factor families, such as *Nkx2, Gata, Mef2, Tbx*, and *Hand* all have been shown to be essential during chamber formation. The functions of these transcription factors ultimately serve to establish chamber identity, so downstream effectors direct appropriate cell differentiation linked to the anatomical position within the heart. Cardiac chamber expansion also requires proteins that drive cell proliferation, which include both transcription factors (Holtz and Misra, 2008; Miano *et al*., 2004; Niu *et al*., 2005; Parlakian *et al*., 2004), as well as mechanotransducers (Granados-Riveron and Brook, 2012). It is not surprising that there are continued requirements for transcription factors during the chamber formation and expansion stages, given the essential role of transcriptional networks in directing cell fate. However, links between mechanotransduction and chamber formation are perhaps more surprising, and underscore the developmental need for cardiac function to propagate the continued cardiac developmental programme.

## TRABECULATION

**Figure 2:**
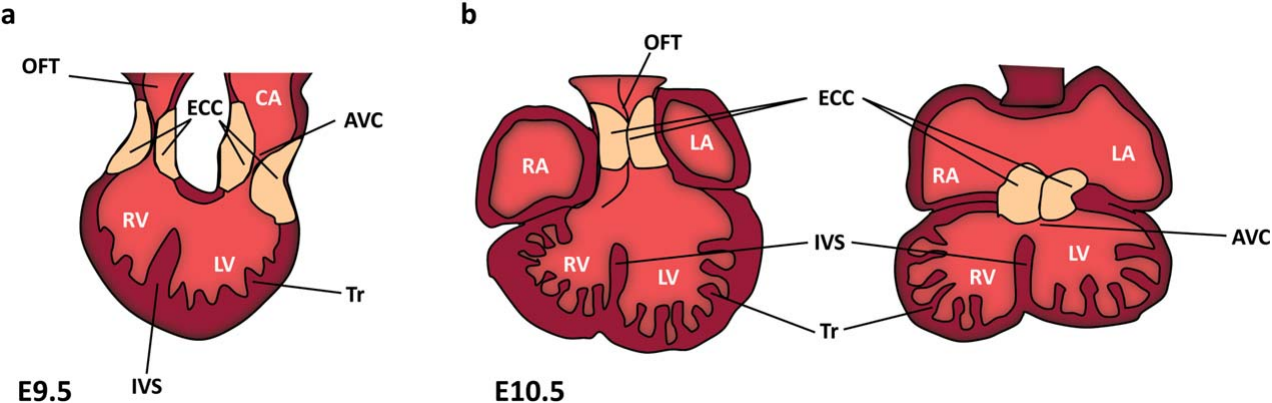
Ventricular trabeculation and endocardial cushion development. Schematic representation of the E9.5 looping mouse heart (a) with location of the outflow tract (left) and atrioventricular (right) endocardial cushions (ECC). Two different cross-sections through the E10.5 mouse heart (b) showing the midgestational location of the OFT cushions (left) and atrioventricular cushions (right). AVC = Atrioventricular canal, CA = Common atrium, IVS = Interventricular Septum, LA = Left Atrium, LV = Left Ventricle, OFT - Outflow Tract, RA = Right Atrium, RV = Right Ventricle, Tr = Trabeculae.

Interactions between the endocardium and myocardium are critical during cardiac development to form essential specialized structures such as ECCs, valves, and trabeculae (Fig. 2). Trabeculation begins around E9.0 in mice with the migration and recruitment of ventricular myocardial cells into the cardiac jelly between the myocardium and endocardium (Samsa *et al*., 2013). Cells then proliferate and differentiate (earlier than in compact myocardium) forming long thin projections into the endocardium which increase surface area available for oxygen uptake, help to prevent inappropriate blood flow between cardiac chambers and provide the contractile force to support the growing mouse heart from E9.5 to E14.5 (Samsa *et al*., 2013). During compaction, these trabeculae collapse leaving shorter thicker projections to support cardiac structure and contraction during development and through adult life (Risebro and Riley, 2006).

Null mutations of *Nrg1*, *ErbB2*, and *ErbB4* all display similar phenotypes of poorly trabeculated ventricles and lethality between E10.5 and E11.5 in mice (Gassmann *et al*., 1995; Lee *et al*., 1995; Meyer and Birchmeier, 1995). Endocardial Nrg1 signaling mediates ligand-dependent heterodimersation of ErbB2 and ErbB4 within the heart myocardium. These dimers become phosphorylated and enable docking of cytoplasmic proteins involved in signal transduction (Negro *et al*., 2004). Mice null for *Efnb2*, encoding EphrinB2, and its specific receptor *EphB4* both display failure to form ventricular trabeculae and lethality between E10.5 and E11.0 due to defective signaling between endocardial cells (Gerety *et al*., 1999; Wang *et al*., 1998). These examples suggest that intercellular signaling is important in trabeculation.

Signaling within the trabeculae themselves also regulates their growth. *Bmp10* is transiently expressed within the trabecular myocardium between E9.0 and E13.5. *Bmp10* null mice have thin hypoplastic ventricular walls and demonstrate arrested growth of trabeculae and lethality around E10.5 (Chen *et al*., 2004). This phenotype may be attributed to essential interactions between Bmp10, cardiogenic transcription factors Nkx2–5, Mef2c and Tbx20, and the trabeculae-specific cell cycle regulator Cdkn1c (Chen *et al*., 2004; Kochilas *et al*., 1999; Zhang *et al*., 2011). It has been elegantly shown that Bmp10, Nrg1, and EphrinB2 signaling all act downstream of Notch signaling during trabeculation (Grego-Bessa *et al*., 2007). However, the lethality of Notch pathway mutants has yet to be linked to trabeculation defects.

The formation of large specialized structures such as ventricular trabeculae concomitantly requires genes encoding proteins with specialized function beyond signaling and cell programming. For example, the cardiac jelly must contain a permissive microenvironment to allow the passage of signaling molecules and which trabeculae can physically negotiate. Adamts1 is a matrix metalloproteinase that degrades cardiac jelly components including Versican (Kern *et al*., 2006; Kuno *et al*., 2000; Rodriguez-Manzaneque *et al*., 2002). Although not fully penetrant, deletion of *Adamts1* in mice results in ∼50% embryonic lethality likely due to uncontrolled trabecular growth (Stankunas *et al*., 2008). The requirement for genetic functions beyond cell signaling and programming is a characteristic shared with ECC formation.

## ECC FORMATION

The mature adult heart contains pulmonary and aortic valves in its arterial pole and mitral and tricuspid valves separating atria from the ventricles in the left and right sides, respectively. Valvulogenesis begins with the formation of ECCs during cardiac looping, which persist through midgestation (Fig. 2). ECC formation takes place in the OFT and AVC beginning with localized production of extracellular matrix (ECM) by myocardium forming the cardiac jelly (Miquerol and Kelly, 2013). These ECM proteoglycans are hydrophillic and the volume of matrix produced causes tissue swelling, forming cushions to prevent the inappropriate backflow of blood. Myocardial and endocardial signals then induce activation of endocardial cells, allowing these cells to break interaction with the neighboring endocardium and invade the cardiac jelly. Migrating cells then undergo EMT to populate ECCs with mesenchymal cells (Combs and Yutzey, 2009; Person *et al*., 2005; Schroeder *et al*., 2003). The ECCs then undergo ECM remodeling and elongate to form the heart valve leaflets (Chakraborty *et al*., 2009).

Similarly to trabeculation, a permissive microenvironment must be present to allow ECC formation. Cushions are absent in *Has2* and *Vcan* null mice due to absence of Hyaluronan and Versican, respectively, in both cases causing lethality from E9.5 to E11.5 (Camenisch *et al*., 2000; Mjaatvedt *et al*., 1998; Yamamura *et al*., 1997). Hyaluronan and Versican interact with fellow ECM components and initiate signaling cascades (Aspberg *et al*., 1999; Binette *et al*., 1994; Hirose *et al*., 2001; Kawashima *et al*., 2000; Kern *et al*., 2006; LeBaron *et al*., 1992; Lionetti *et al*., 2010; Maioli *et al*., 2010; Zhang *et al*., 1998). If Hyaluronon and Versican act as links between the ECM and intracellular signaling cascades, this could explain why they are so essential for ECC formation. Hyaluronan has also been associated with cell migration which precedes EMT since Has2 null AVC explants could be rescued by Hyaluronan treatment, which induces phosphorylation of ErbB2 and ErbB3, thus rescuing cushion mesenchyme formation (Camenisch *et al*., 2002).

In valvulogenesis genes that control cell proliferation become especially important during valve elongation, particularly in endocardial derived ECC cells. The transcription factor *Nfatc1* is highly expressed in valve endocardial cells and is essential for valve and septum formation; in mice with nonfunctional *Nfatc1* cardiac valves remained immature (de la Pompa *et al*., 1998; Ranger *et al*., 1998). Nfatc1 supports valvulogenesis by promoting valve endocardial proliferation and simultaneously inhibiting EMT, ensuring a large enough endocardial population for proper valve growth (Wu *et al*., 2011). Endocardial expression of *Tbx20* promotes cell proliferation in valvulogenesis, as well as Wnt/β-catenin signaling, but is dispensable for EMT (Cai *et al*., 2013).

Classical intercellular signaling is important in ECC formation. All Notch receptors and ligands are expressed in mouse endocardium during the onset of ECC formation (Timmerman *et al*., 2004). Deletion of Notch pathway components *Rbpjk* and *Notch1* result in collapse of ECCs due to failure of cells to undergo EMT and exhibit lethality at E10.5. The majority of evidence supports ECC explant culture experiments suggesting that Rbpjk and Notch1 signal via Snai1. Snai1 in turn represses *VE-Cadherin* expression, allowing endocardial cells to destabilize adherens junction cell contacts, delaminate, and invade the cardiac jelly to undergo EMT (Saad *et al*., 2010; Timmerman *et al*., 2004). However, Notch1 also appears to act downstream of and converge with Bmp2 signaling during cushion cell invasion and EMT, forming a complex regulatory web between the myocardium and endocardium. Myocardial Bmp2 upregulates endocardial *Notch*, which in turn downregulates endocardial, but not myocardial, *Bmp2* (Fischer *et al*., 2007; Kokubo *et al*., 2005; Luna-Zurita *et al*., 2010).

## TRABECULATION AND ECC DEVELOPMENT DISPLAY OVERLAPPING ESSENTIAL FUNCTIONS

Genes with essential functions during trabeculation and ECC formation reveal an overlapping requirement for ECM and basement membrane proteins (Costell *et al*., 2002; Sasse *et al*., 2008) during these processes. The increasing importance of the extracellular environment as cardiac development progesses is apparent, as animal mutants with perturbations in the production or function of molecules needed for the ECM resemble human congenital heart defects (Lockhart *et al*., 2011). Although ECM is found within all the structures of the developing heart, and is present in the form of cardiac jelly in the early heart tube (Markwald *et al*., 1977), the ECM is particularly important for the formation of endocaridal cushions (Lockhart *et al*., 2011) and trabeculation. Mutants lacking various matrix molecules or enzymes needed for matrix protein production display thin myocardial walls that lack trabeculae in addition to ECC defects (Camenisch *et al*., 2002; Camenisch *et al*., 2000; Mjaatvedt *et al*., 1998; Yamamura *et al*., 1997), highlighting the similar processes needed to create these disparate structures. Despite this, the diversity in the signaling cascades required in the processes of ECC formation and trabeculation provides for distinctions between these two events during cardiac development.

## EPICARDIAL DEVELOPMENT

**Figure 3:**
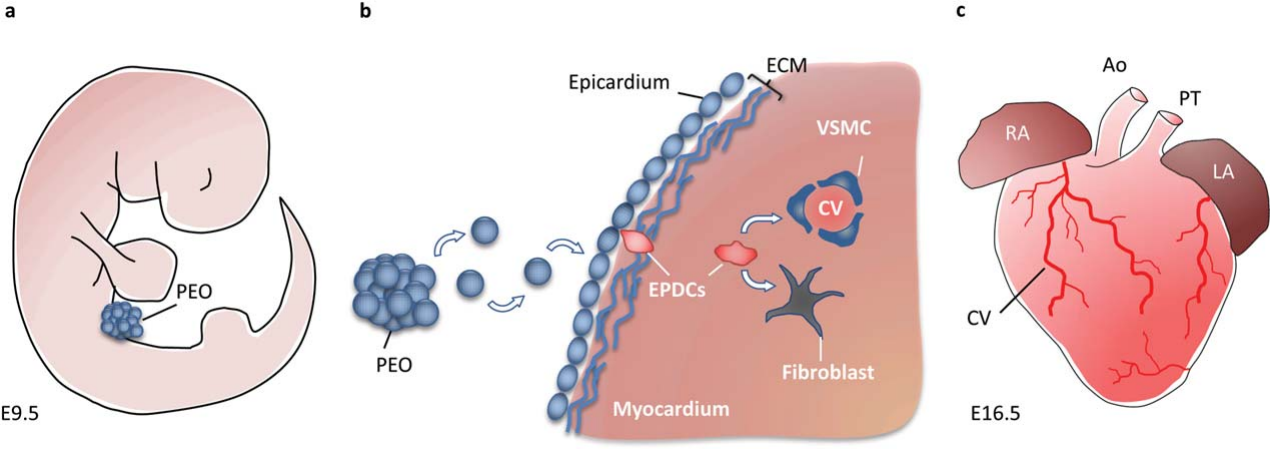
The PEO contributes to CV formation. (a) The proepicardial organ (PEO) is a transient structure that forms near the sinus venosus of the postlooped embryonic heart at approximately E9.5. (b) Cells within the proepicardial organ translocate across the pericardial cavity and adhere to the myocardium to form an epithelial sheet that envelopes the developing heart, called the epicardium, by E11.0. Later in development, a sub population of epicardial cells undergo epithelial to mesenchymal transition, allowing them to migrate through the subepicardial ECM and invade the myocardium. These EPDCs differentiate into VSMC and cardiac fibroblasts, and are essential for the formation of the mature CV, as observed on the ventricular surface of the E16.5 heart (c). Ao = Aorta, LA = Left Atria, PT = Pulmonary Trunk, RA = Right Atria.

In mice, the PEO originates from the pericardial mesothelium covering the pericardial surface of the septum transversum below the heart near the sinus venosus, where a villous structure protruding toward the looped heart is formed (reaching maximal size around E9.5; Fig. 3). In mammals, cells from this structure are thought to undergo vesicular budding into the pericardial space followed by adherence to the myocardium (E9.5–10). However, in rats and mice the PEO and dorsal surface of the heart interact directly, allowing cells to adhere to myocardium via ECM or adhesive protein tissue bridges (Nesbitt *et al*., 2006; Rodgers *et al*., 2008). Once the cells have reached the myocardial surface they proliferate and migrate laterally to eventually envelop the heart (completed by E11). The PEO then ceases to exist and PEO-derived cells become differentiated to form the epicardium. The epicardium makes cellular contributions to multiple lineages of the myocardium via delamination, EMT and myocardial invasion. The epicardium is also the main source of cardiac fibroblasts (reviewed in (Souders *et al*., 2009), prior to EMT the whole epicardium expresses *Tcf21*, which is necessary but not sufficient for fibroblast fate specification (Acharya *et al*., 2012). In addition to cellular contributions, the epicardium is also a potent source of trophic factors that stimulate myocardial growth, differentiation, and coronary artery formation (Cai *et al*., 2008; Lie-Venema *et al*., 2007; Smart *et al*., 2007; Sucov *et al*., 2009; Zhou *et al*., 2008a).

Migration of epicardial progenitors to and along the myocardium is an essential requirement for cardiac development, and correct cell polarity is required for this process. *Par3* null mouse embryos suffer lethality from E10.5 to E12.5 because a loss of cell polarity results in a failure to form the vesicles that bud from the PEO, causing subsequent absence of epicardium in mouse hearts. These defects were identified through aberrant intracellular localization of Pard6β, Prkcι, and Ezrin (Hirose *et al*., 2006). Additionally, *Pdpn* null mice demonstrate lethality during midgestation due to failure to break cell–cell contact and subsequent decreased delamination of cells from the PEO associated with inability to remove E-Cadherin (Mahtab *et al*., 2008).

Once cells have migrated from the PEO, they must adhere to the myocardium. Mice null for the myocardially expressed cell adhesion molecule *Vcam1* display lethality around E11.5–E12.5 exhibiting absent epicardium with subsequent thinner compact myocardium accompanied by pericardial haemorrhage (Kwee *et al*., 1995). Mutations in *Itga4*, coding for α_4_ integrin, mirror these defects as α_4_β_1_ integrin, along with its two major ligands Vcam1 and Fibronectin, are essential for epicardial adhesion both initially and long-term (Sengbusch *et al*., 2002; Yang *et al*., 1995). The proepicardium marker gene Wilms tumor suppressor 1 (Wt1) regulates expression of *Itga4*; embryos deficient in α_4_integrin resemble Wt1 knockout embryos (Kirschner *et al*., 2006).

Once attached, epicardial cells direct myocardial cell proliferation, maturation, mechanical/electrical coupling, and cellular alignment to maximize contractile force (Weeke-Klimp *et al*., 2010). Wt1, which in the heart is specific to the epicardium, controls synthesis of the highly potent morphogen retinoic acid (RA) through *Raldh2* expression (Guadix *et al*., 2011; Kirschner *et al*., 2006). *Raldh2* null mice demonstrate lethality by E10.5 with defective chamber development, myofilament gene expression, cardiomyocyte differentiation and mechanical looping defects (Niederreither *et al*., 2001), suggesting that epicardially synthesized RA is needed for myocardial development. Within the epicardium, RA signaling appears reliant on retinoid receptor Rxrα. *Rxrα* null mice exhibit lethality from E13.5 to E17.5 due to ventricular hypoplasia and reduced trabeculation that appears to stem from failure in epicardial adhesion to the myocardium (Sucov *et al*., 1994). Subsequent studies on *Rxrα* null mice have showed delayed cellular migration from the PEO, elevated apoptosis of PEO cells, increased myocardial fibronectin and decreased epicardial Vcam1 causing increased area of subepicardial spaces between epicardium and myocardium (Hoover *et al*., 2008; Jenkins *et al*., 2005). Nonlocal sources of RA are also essential; RA signaling in the liver controls *Epo* expression, whose product travels to the heart and stimulates myocardial cell proliferation (Brade *et al*., 2011). Expression of its receptor, *Epor*, is found in the epicardium while expression *Epo* itself is absent from the heart; both are essential genes (Koury *et al*., 1988; Wu *et al*., 1999).

Perhaps the transcription factor that has been most characterized with regards to its function in the epicardium is Wt1. Wt1 has been shown to promote epicardial EMT via transcriptional regulation of *Snai1* and *E-cadherin* (Martinez-Estrada *et al*., 2010) and regulate RA signaling through transcriptional activation of *Raldh2* (Guadix *et al*., 2011). Subsequent to epicardial EMT, epicardial-derived cells contribute to the developing coronary vasculature, which is critical for late-gestation cardiac function (Dong *et al*., 2008; Perez-Pomares and de la Pompa, 2011).

## ESSENTIAL GENES FOR EPICARDIAL FUNCTION

The development of the epicardium from the PEO coincides with an expansion in the complexity of the developing heart, and concomitantly, an increase in the number of essential genes. As the epicardium migrates onto the heart surface during development to envelop the developing myocardium, cellular interactions between the epicardium and myocardium are formed. Genes mediating these interactions are essential for cardiac development (Kwee *et al*., 1995; Sengbusch *et al*., 2002; Yang *et al*., 1995). Yet the epicardium is also a source of signals to promote ventricular development, and, therefore, a diverse array of molecules is essential for epicardial function. Many mutants with epicardial defects display a thin ventricular myocardium, indicating a link between epicardial function and myocardial cell proliferation or compaction (Brade *et al*., 2011; Mahtab *et al*., 2008; Niederreither *et al*., 2001; Sucov *et al*., 2009; Weeke-Klimp *et al*., 2010). There are both signaling molecules and transcription factors that interconnect epicardial and myocardial development. Following epicardial EMT, epicardial derived cells (EPDCs) contribute to the forming coronary vasculature (Perez-Pomares and de la Pompa, 2011; Riley and Smart, 2011; Ruiz-Villalba and Perez-Pomares, 2012; von Gise and Pu, 2012). As many processes in epicardial formation are conserved and essential throughout embryogenesis, loss of these genes often results in early lethality. Embryos that survive to midgestation exhibit lethality because defects in epicardial cell function will cause defects in coronary vessel (CV) formation.

## CARDIAC NEURAL CREST CELLS AND PHARYNGEAL ARCH ARTERY FORMATION

Neural crest cells (NCCs) migrate from the dorsal neural tube between the midotic placode and the caudal boundary of the third somite into the pharyngeal arches and OFT where they eventually form part of the OFT septum (Jiang *et al*., 2000). The pharyngeal arch arteries (PAAs) emerge proximally from the aortic sac and distally from the descending aorta to form continuous arteries during early to midgestation. The PAAs are numbered 1–6, although the fifth artery is thought to be rudimentary or absent, and form sequentially. The first PAA emerges by E9.0, the second by E9.5, and the third, fourth, and sixth between E9.5 and E10.0. The PAAs are initially formed from mesoderm-derived endothelial cells and rely on cranial and cardiac (from rhombomeres 1, 2, and 4, and 6–8, respectively) NCC contributions for their maintenance and remodelling. Specifically, cranial NCCs contribute to the first and second pharyngeal arches including the first and second PAAs, while the cardiac NCCs contribute to the third, fourth, and sixth pharyngeal arches including the third, fourth, and sixth PAAs. Beginning at ∼E11.0, the right-sided PAAs regress and are lost, leaving the left-sided PAAs to be remodelled and become integral components of the vascular system. For example, the fourth PAA becomes the arch of the aorta from which the left subclavian and common carotid arteries emerge. Unsurprisingly, the precise spatiotemporal regulation required for PAA development provides ample opportunity for defects to manifest themselves when pathway components are altered or absent (Graham, 2003; Hiruma *et al*., 2002; Snider *et al*., 2007).

Similar cardiac inductive and migratory processes are required in mouse NCCs as in PHF and SHF as shown by mutations of members of the Bmp, Fgf, and Wnt pathways (Kubota and Ito, 2000; Nie *et al*., 2008; Schleiffarth *et al*., 2007; Song *et al*., 2010; Tang *et al*., 2010; Zhang *et al*., 2010a). Genes specific to NCCs (in the context of cardiogenesis) include *Sox9*, *Snai2*, and *Foxd3*, with the former two influencing EMT and differentiation and the latter influencing NCC induction (Cheung *et al*., 2005). Interactions between the SHF and NCCs appear to be mutually important in regulating EMT and NCC migration; this is revealed in mutants with loss of function of *Pax3* or components of Fgf signaling (Bradshaw *et al*., 2009; Park *et al*., 2008; Zhang *et al*., 2008). *Foxd3* and *Pax3* interact genetically to allow cell survival in cardiac NCC progenitors, although the nature of this interaction is unknown (Nelms *et al*., 2011). Unsurprisingly, the majority of defects observed in the cardiac NCCs affect OFT septation and alignment, as observed in embryos with *Pax3* loss of function or NCC-specific deletion of *N-cadherin* (Bradshaw et al., 2009; Luo et al., 2006). These defects arise from wide ranging aspects of NCC migration, specification, proliferation, and EMT (Snider *et al*., 2007; Vincent and Buckingham, 2010).

Tgfβ signaling also appears to be an integral component of PAA development, suggesting similarities between PAA and normal vascular development. *Tgfβ2* null mice experience inappropriate apoptosis of the fourth PAA, which contributes to the perinatal lethality observed in these mice (Molin *et al*., 2002; Molin *et al*., 2004). Similarly, NCC-specific deletion of Tgfβ receptor *Alk5* results in abnormal PAA remodelling due to aberrant apoptosis of neural crest derived cells (Wang *et al*., 2006). Function of the transcription factor Mrtfb, which is needed for vascular smooth muscle cell (VSMC) differentiation, is mediated by SMAD2 (Li *et al*., 2005; Xie *et al*., 2013). Mice null for transcription factor *Mrtfb* experience lethality by E14.5, displaying widespread haemorrhaging as well as defects in PAA remodeling and VSMC differentiation (Oh *et al*., 2005).

Bmp signaling is critical for the induction and/or EMT of NCCs that contribute to PAAs; mice with NCC-specific deletion of Smad4 are lethal by E12 with reduced expression of NCC markers including *Tfap2a*, *Sox9*, and *Msx1* and *2*. This causes apoptosis of both neural crest and nonneural crest derived cells within the developing pharyngeal arches (Nie *et al*., 2008). Similarly, NCC-specific conditional mutants of *Bmpr1a* display hypoplasia of OFT cushions, inappropriate backflow of blood and lethality (Nomura-Kitabayashi *et al*., 2009).

## ESSENTIAL GENES FOR CARDIAC NCC FUNCTION AND PAA FORMATION

Cardiac NCC function, migration and the subsequent formation of the PAAs shares many of the same processes as cardiac chamber or epicardium formation and maturation. In all these events cellular differentiation, migration, and adhesion are indispensable, as are the genes that control these processes. As a result, it is not surprising that familiar pathways, such as the Fgf, Bmp, and Tgfβ signaling pathways, are needed in cardiac NCC and PAA development. Where genes specific to cardiac NCC and PAA fit into established pathways is less well characterized, but it is clear that other signaling pathways and transcription factors are also essential, and these may be poorly understood. For instance, mice null for genes needed to produce endothelin 1, such as *Edn1* and *Ece1*, exhibit inappropriate persistence and/or regression of the PAAs during remodelling (Yanagisawa *et al*., 1998). It is likely that many genes and pathways which contribute to defects in PAA remodelling are yet to be discovered, given the complexity and lack of complete understanding of this developmental process.

## SARCOMERE FORMATION

As the heart begins to circulate blood, structural genes, and the genes that control them, become essential. Contraction of muscle fibers including those of the heart is achieved through the actions of the sarcomere, the functional unit of contractility, which contains contractile, structural and regulatory proteins. Fully formed sarcomeres are visible from E9.0 in mice and are large multiprotein complexes (Craig and Padron, 2004), which retain their structural integrity despite having to constantly and rapidly contract/relax and switch on/off to meet the demands of the heart. These structures allow transduction of force throughout the cell to the ECM and sense mechanical stretch (Boateng and Goldspink, 2008). In contrast to the transcriptional and signaling pathways that direct many of the events of cardiac development, the functions encoded by genes lethally disrupting sacromere function do not show much overlap with other groups of cardiac essential genes.

**Figure 4:**
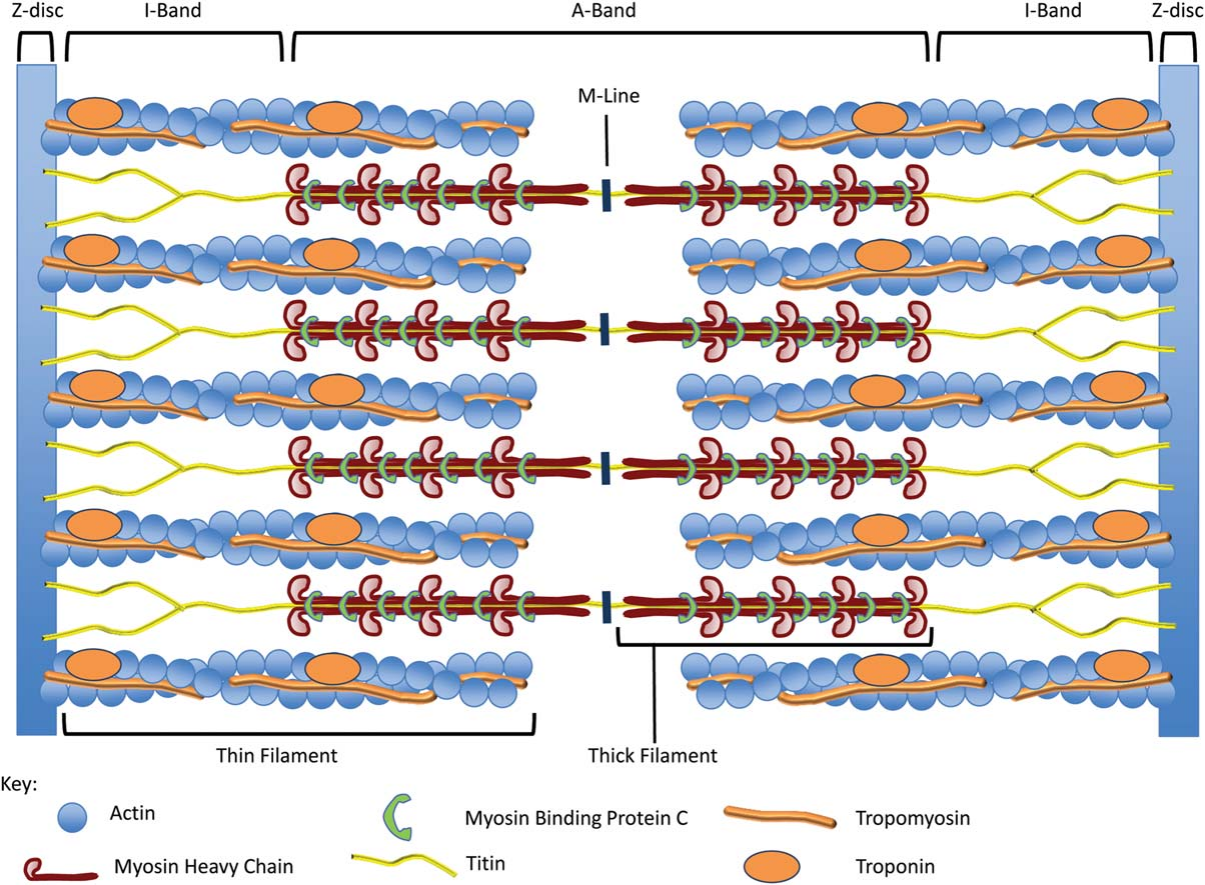
Sarcomere structure. Schematic representation of the mouse sarcomere showing the relevant banding pattern according to its electron microscopic appearance. Thin filaments are comprised of two interacting helically organized F-Actin polymeric chains along which Tropomyosin polymers lie in the grooves. Troponin complexes are found at set periods along the thin filament. Thick filaments are comprised of over 300 Myosin II molecules arranged into filament bundles and many interacting nonmyosin proteins. Individual molecules of the giant protein Titin span from Z-disc to M-line. Adapted from (Morimoto, 2008).

The structure of the sarcomere is understood in terms of its longitudinal appearance via electron microscopy in relaxed state (Fig. 4). The sarcomere is bordered at each end by the dark Z-disc of ∼0.1 µm length. The Z-disc bisects the I-band, which is ∼1 µm long, is shared between adjacent sarcomeres and is made up of thin filaments (10 nm diameter). The A-band lies between I-bands and is predominantly made up of thick filaments (15 nm diameter) with a slight overlap of thin filaments and is ∼1.6 µm length. At the centre of the A-band is the higher density H-zone appearing lighter with the M-line in turn at its centre. In cross section, where thick and thin filaments overlap, thin filaments are hexagonally arranged around thick filaments (Craig and Padron, 2004).

Several structural sarcomeric proteins perhaps unsurprisingly have proven to be essential for cardiac development. At up to 3.7 MDa, Titin is the largest known protein and spans from the Z-disc to the M-line within the sarcomere (LeWinter *et al*., 2007). Due to the vast size and volume of interacting partners, Titin has been studied on a modular basis. Evidence has suggested Titin regulates sarcomeric length, plays important roles in cardiac stress responses and contributes to diastolic properties of the heart (Kontrogianni-Konstantopoulos *et al*., 2009; Linke, 2008). A truncation mutant of *Ttn* (which encodes Titin) thought to cause degradation of Titin causes embryonic lethality at ∼E9.0 due to defective sarcomeric formation (Gramlich *et al*., 2009). An M-line deficient mutant of Titin led to developmental delay by ∼E11.0 followed by embryonic lethality due to lateral growth defects in sarcomeres and their consequent disassembly (Weinert *et al*., 2006).

The thick filaments of sarcomeres are made up of members of the Myosin II superfamily, as well as other nonmyosin proteins. Each Myosin II molecule comprises two myosin heavy chains which form an insoluble coil via their tail domains and two pairs of myosin light chains which attach to the neck domain of each heavy chain, respectively, leaving the head domains in close proximity (Craig and Padron, 2004; Craig and Woodhead, 2006). When in a high Ca^2+^ environment the head domains interact with actin and ATP, providing the energy for contraction, which occurs through the slippage of cross bridges between myosin and actin (Huxley and Hanson, 1954). Given the important structural and enzymatic properties of myosin heavy chains, it is unsurprising that they are essential genes for cardiogenesis. For instance, deletion of Myosin II member *Myh6* causes embryonic lethality from E11.0 to E12.0 (Jones *et al*., 1996). Deletion of atrial or ventricle-specific Myosin light chains *Myl7* or *Myl2* results in embryonic lethality at E11.5 and E12.5, respectively, due to sarcomeric disorganization accompanied by morphological cardiac defects (Chen *et al*., 1998; Huang *et al*., 2003).

The thin filaments of sarcomeres consist of a double helix of two F-Actin polymeric chains, along which are bound two parallel Tropomyosin polymers and periodic Troponin complexes (reviewed in Craig and Padron, 2004). In a Ca^2+^ rich environment, inhibition of contraction by Troponin is lifted, allowing Actin-Myosin interaction, ATPase activity and movement of the thin filaments along the thick filament towards the M-line (Galinska *et al*., 2010; Galinska-Rakoczy *et al*., 2008; Huxley and Hanson, 1954; Lehman *et al*., 2009; Solaro, 2010). *Tnnt2* encodes the cardiac-specific isoform of Troponin subunit TnT of which null mice demonstrate lethality at ∼E10.0 with absent sarcomeres and absent heartbeat (Nishii *et al*., 2008). Tropomodulin prevents Actin depolymerization and elongation at the end of appropriately sized thin filaments and also interacts with Tropomyosin. Unsurprisingly, *Tmod1* null mice exhibit lethality by ∼E9.5, with an absence of sarcomeres and defective cardiac looping (McKeown *et al*., 2008). Mice null for cardiac Actin *Actc1* also experience 56% embryonic lethality rate in late gestation due to sarcomeric disorganization and presumptive compromised cardiac function (Kumar *et al*., 1997).

A number of nonstructural genes are essential for sarcomere development; these are genes needed to induce proper sarcomere formation or regulatory pathway components enabling sarcomeres to respond to stress, developmental stimuli or to maintain sarcomeric function. These genes by necessity have more diverse functions than structural sarcomeric functions. Cardiac-specific light chain kinase, which phosphorylates Myosin regulatory light chain 2 ventricular/cardiac isoform (Mlc2v), is essential for sarcomere assembly (Seguchi *et al*., 2007) and appears to be under the control of Nkx2–5 (Chan *et al*., 2008). Calreticulin is an endoplasmic reticulum protein which binds Ca^2+^ and regulates its intracellular homeostasis (Michalak *et al*., 1999). *Calr* null mice demonstrate embryonic lethality from mid to late gestation with ventricular hypoplasia, random sarcomeric orientation, sarcomeric waviness, and thinning (Lozyk *et al*., 2006; Mesaeli *et al*., 1999).

## SARCOMERE FUNCTIONAL ESSENTIALITY IS DISTINCT

In contrast to the transcriptional and signaling pathways that direct many of the events of cardiac development, the functions encoded by genes lethally and exclusively disrupting sacromere function do not show much overlap with other groups of cardiac essential genes. From midgestation, the mammalian embryo requires cardiac contractility to pump blood throughout the cardiovascular system, delivering nutrients to embryonic cells. Impaired cardiac function as a result of disordered or functionally incomplete contractile apparatus assembly leads to embryonic lethality. Structural proteins that allow myofilament assembly and function are required within the sarcomere to form the contractile units of the developing heart. The specific mechanical and structural role of sarcomere proteins makes it unsurprising that there is a functionally distinct group of genes displaying essentiality during sarcomere assembly, which are not involved in other aspects of cardiac development.

## CONCLUSIONS

Complete functional annotation of mammalian genomes is beginning to appear as a realistic possibility (Dolgin, 2011; Skarnes *et al*., 2011; White *et al*., 2013). The increasingly systematic nature of random mutagenesis and mutation identification, targeted mutagenesis/cloning and identification of epigenetic modifications along with increasing computational power and variety of bioinformatic approaches are facilitating these efforts. Systems biological approaches can be used to model metabolic pathways, dynamics of specific cell behavior under varying stimuli, disease causing aberrations in functional networks and in screening for potential therapeutic agents (Chen *et al*., 2012; Lambrou *et al*., 2012; Nagasaki *et al*., 2011; Tan *et al*., 2013). These approaches can also demonstrate the consequences of nonsynonymous DNA polymorphisms on protein interactions and identify mutations leading to development of diseases (Reimand and Bader, 2013; Yates and Sternberg, 2013a). Perhaps most relevantly, analysis of the characteristics of genes that confer essentiality or disease-susceptibility will lead to insights into the genetic networks required for cellular functions (Yates and Sternberg, 2013b). As systems biological data accumulates it may be possible in the near future to scale these networks up to construct a hierarchy for the functional importance of genes or domains at the whole mammalian genome scale.

Efforts to functionally annotate the mammalian genome have increased the identification of essential genes. Recent experimental studies support the prediction that approximately 40% of mammalian genes are essential (White *et al*., 2013). Interlinked with the identification of essential genes is the discovery of new developmental functions, as essential genes inherently are required during development. The in utero demand for a functional cardiovascular system further underscores the integration of gene essentiality and cardiac development. During the process of heart formation genes with diverging functions become essential. An analysis of the functional diversity of cardiac essential genes throughout development therefore reveals the changing cellular and biochemical demands of the developing heart. As more essential genes are annotated, links between gene function and specific cardiac developmental events will expand our knowledge of genetic factors that may contribute to cardiac developmental defects including congenital heart disease.
